# Usage of glaucoma-specific patient-reported outcome measures (PROMs) in the Singapore context: a qualitative scoping exercise

**DOI:** 10.1186/s12886-018-0803-5

**Published:** 2018-08-14

**Authors:** Owen Kim Hee, Zheng-Xian Thng, Hong-Yuan Zhu, Ecosse Luc Lamoureux

**Affiliations:** 1 National Healthcare Group Eye Institute, Tan Tock Seng Hospital, S308433, Singapore, Singapore; 2 Health Services Research, Singapore Eye Research Institute, Singapore, Singapore

**Keywords:** Glaucoma, Patient-reported outcome measures, Questionnaire

## Abstract

**Background:**

Despite the increasing emphasis on the role of glaucoma-specific patient-reported outcome measures (PROMs) as relevant outcome measures for the impact of glaucoma and its intervention on patients' daily lives, the feasibility of implementing PROMs in the routine clinical setting in Singapore remains undefined. We aim to evaluate the comprehensibility, acceptability, and relevance of four glaucoma-specific PROMs at healthcare professionals' and patients' level in a Singapore context.

**Methods:**

Sixteen ophthalmic healthcare professionals and 24 glaucoma patients, with average age 60 years (SD = 15), were invited from a tertiary hospital in Singapore. Semi-structured interviews were conducted to explore participants’ perceptions on the content and administration of four glaucoma-specific PROMs - the Glaucoma Quality of Life-15, Glaucoma Symptom Identifier, Independent Mobility Questionnaire and Treatment Satisfaction Survey of Intra-ocular Pressure. Semi-structured interviews were hand transcribed, and analysed thematically. Each participant filled out a feasibility survey at the end of interview.

**Results:**

79% of glaucoma patients and 94% of glaucoma healthcare professionals felt selected PROMs relevant to patients. 63% of glaucoma patients and 50% of healthcare professionals felt that selected PROMs were sufficiently comprehensive for clinical use. 46% of glaucoma patients and 56% of healthcare professionals felt selected PROMs were user-friendly.

**Conclusions:**

Using PROMs in the Singapore clinical setting receives promising support from both healthcare professionals and patients. The identified potential barriers tailored to Singapore clinical setting will help successful implementation of PROMs into routine clinical care.

## Background

Important aim of quality health care is the patient-centeredness and a focus on patient experience [1]. However, healthcare professionals have traditionally defined “successful glaucoma treatment” based on objective clinical endpoints such as lowering of intraocular pressure (IOP) and slowing or stopping visual field deterioration [2]. These clinical measures may result in more disease-centric care as opposed to more patient-centric care which requires a more holistic view of care delivery, since they only surrogate measures of an effective treatment paradigm, and cannot fully capture the actual impact of glaucoma and its treatment on patients’ daily lives [3, 4], considering the chronic progressive nature of glaucoma and its potential to substantially and negatively affect patient’s daily functioning [5]. Furthermore, insights into how glaucoma affects patients’ lives might provide a means of tailoring treatment strategies to meet the individual’s specific need [5].

The increasing realization that clinical parameters alone are inadequate to assess health outcomes has resulted in the widespread use of patient-reported outcome measures (PROMs) [6]. PROMs are a series of standardised and validated questions self-reported by patients to assess their perspectives on the impact of diseases and treatment on their own health status, well-being and functioning [6–8]. Especially disease-specific PROMs, as the gold standard for relevant endpoint measures of patients’ subjective experiences, are important to clinicians as feedback on the care they have provided and for assessing the quality of care provided by healthcare services. PROMs have been widely used as effectiveness endpoints for approved drug labels in the United States [7], and outcome assessment in clinical guideline development (e.g., for pain management [9], dialysis treatment [10], and screening for prostate cancer [11]), as well as applied in the context of national audits [12], clinical governance and quality assurance [13], and integrated into routine clinical practice [14] and managing the performance of healthcare providers [15].

However in glaucoma, most attempts to measure health improvement from treatment have largely been in clinical research studies on selected population and much less frequently in routine clinical practice [16, 17]. Also, there is a plethora of PROMs with considerable heterogeneity amongst them, such as differing in terms of how the answers are scored, as well as the number, nature and the wording of the questions asked. The quality of these PROMs instruments also varies considerably in terms of their validity and reliability [16, 18]. Moreover, although most of glaucoma-specific PROMs have been constructed in accordance to basic psychometric principles [19], a conceptual framework building on patient views is absent in more than 50% of instruments [16]. The ethos of PROMs is to gauge patients’ assessment on their own health status and health-associated quality of life*.* So arguably, the lack of patient input in the development process of these PROMs has been cited as a methodological shortcoming. An appropriate PROM should be supported by published evidence presenting that it is acceptable to patients, as well as valid, reliable, and responsive (sensitive to change) [20]. Additionally, feedback from healthcare professionals on PROMs use is also essential addition to any PROM development [21].

In Singapore, there has also been growing interest in exploring the use of PROMs in routine glaucoma care. However, most, if not all available PROMs have been developed in the context of largely western populations with different healthcare systems [16, 18]. Singapore, on the other hand, is a multi-ethnic country with a majority population of Chinese (74.2% of the resident population), with substantial Malay (13.2%), Indian minorities (9.2%) and other ethnicities (3.3%). Little is known on the use of these PROMs in a multi-ethnic Asian country like Singapore with a different healthcare system.

Hence, our study aimed to qualitatively gauge the relevance, comprehensiveness and acceptability of using four glaucoma-specific PROMs, namely: the Glaucoma Quality of Life-15 (GQL-15) [22], Glaucoma Symptom Identifier (GSI) [1], Independent Mobility Questionnaire (IMQ) [23], and Treatment Satisfaction Survey of Intra-ocular Pressure (TSS-IOP) [24], in the routine glaucoma care in the Singapore context. These four glaucoma-specific PROMs were selected based on a systematic review identifying them as the PROMs with the greatest potential for further adaptation and testing in the clinical setting [16]. To achieve our aim, we conducted a semi-structured interview with healthcare professionals and glaucoma patients about their perceptions on the content of one of four selected PROMs instrument and issues relating to the administration. A feasibility survey was performed upon the conclusion of the semi-structured interview.

## Methods

This study was approved by the Institutional Review Board of Singapore National Healthcare Group.

### Literature review

An extensive background literature review in PUBMED database of existing glaucoma specific “patient reported outcome measures” (PROMs) instruments was carried out in March 2014. The search terms used include “glaucoma”, “quality of life”, “patient reported outcome”, “questionnaire”, “survey”, “development” and “validation”. Amongst others, two systemic reviews [16, 18] analysed 33 and 27 PROMs instruments, respectively, and included details on their development and validation. Our study team took into account the authors’ recommendations, availability of the full instrument in English, feasibility of actual administration during clinical practice and underwent multiple roundtable discussions before shortlisting 4 of the most promising glaucoma specific PROMs instrument for use, namely: GQL-15 [22], GSI [1], IMQ [23], and TSS-IOP [24].

### Semi-structured interviews and feasibility survey

To ensure comprehensive viewpoints on the implementation of glaucoma-specific PROMs instruments in daily clinical practice were gathered, both healthcare workers involved in the care of glaucoma patients and glaucoma patients themselves took part in the semi-structured interviews. This purposive sampling process included relevant stakeholders such as consultants, optometrists, ophthalmic nurses and low vision occupational therapists. Patients were recruited to include a variety of glaucoma diagnosis types and severity. All participants were capable of giving informed consent.

These interviews were performed through trained ophthalmic healthcare professionals in English. Each participant was presented with 1 of the 4 selected PROMs instruments. A “think aloud” method was used where participants were encouraged to verbalize their views and perceptions on the items of the instrument. ‘Think aloud’ is one valuable cognitive interviewing technique for gaining insights into participant’s cognitive processes whilst performing a task [25]. During this process, interviewers hand transcribed and verbatim these enunciated views. A topic guide developed based on literature review and clinical experience was used to guide the interview and obtain feedback on not only the content of the PROMs instrument itself but also issues relating to the administration of it. Consideration was given to the power relationship between doctors and patients, with non-doctors who were not directly involved in the patients’ care assigned to conduct the interviews. At the end of the interview, participants were asked to fill out a simple 4 questions feasibility survey devised to gauge the relevance, comprehensiveness and acceptability of the usage of glaucoma specific PROMs instruments in a busy clinical setting.

### Thematic analysis

Two researchers of the study team independently conducted thematic analyses of the interview results. This was followed by further roundtable discussions with the rest of the study team to refine the analysis. The themes that emerged were then assigned to domains considered important to the successful use of glaucoma specific PROMs instrument in day-to-day clinical practice. These domains were developed after extensive literature review [26], research findings and consultation with clinicians and operations staff. Subsequently, the domains were back-mapped onto the 4 PROMs instruments to gain further insight on the strengths and limitations of each individual instrument as a complement to traditional clinical assessment tools of glaucoma patients.

## Results

### Demographics of participants

Table 1 presents participants’ demographic data. A total of 40 participants, consisting of 16 (40%) healthcare professionals and 24 (60%) glaucoma patients, took part in the semi-structured interviews. The majority of the participants was Chinese (66%), followed by and Indian (17%), Eurasian (13%), others (4%), and Malay (0%). Amongst the 16 healthcare professionals, there were four from each of the following groups: glaucoma ophthalmologists, optometrists, ophthalmic nurses and low vision occupational therapists. These were all employees of the same tertiary hospital as the study team. Of the 24 glaucoma patients, 8 each with early, moderate or severe glaucoma based on the Bascom Palmer (Hodapp-Anderson-Parrish) Glaucoma staging system. All could either read or understand English and had no other significant ocular pathology.

**Table 1:** Demographic Characteristics of Patients Recruited

Age (mean ± SD)	60 ± 15 years
Gender	
Male	19 (79%)
Female	5 (21%)
Race	
Chinese	16 (66%)
Malay	0 (0%)
Indian	4 (17%)
Eurasian	3 (13%)
Others	1 (4%)
Employment status	
Fulltime	14 (58%)
Part-time	0 (0%)
Unemployed	10 (42%)
Education level	
Nil	2 (9%)
Primary	1 (4%)
Secondary	8 (33%)
Tertiary	13 (54%)
Monthly income	
Nil	12 (50%)
$1 - $4999	6 (25%)
$5000 - $9999	6 (25%)
> $10,000	0 (0%)
Glaucoma type	
POAG	15 (62%)
PACG	3 (13%)
NTG	4 (17%)
Secondary glaucoma	2 (8%)
Duration of glaucoma (mean ± SD)	7.58 ± 5.95 years
Current management	
Topical medications only	13 (54%)
Topical medications and laser	6 (25%)
Topical medications and surgery	3 (13%)
Surgery only	2 (8%)
LogMAR Visual acuity (better eye) (mean ± SD)	0.17 ± 0.3
LogMAR Visual acuity (worse eye) (mean ± SD)	0.45 ± 0.57
Mean deviation (better eye) (mean ± SD)	−7.72 ± 8.30 (dB)
Mean deviation (poor eye) (mean ± SD)	− 10.8 ± 7.84 (dB)

### Thematic analysis and domain identification

A total of 286 comments were recorded from the semi-structured interviews. Of these, 139 were made by the glaucoma healthcare professionals and 147 by glaucoma patients. Following analysis, the comments were dichotomized into 2 main themes: those relating to the content of the PROMs instrument and those relating to the actual administration of the instrument. Sub-themes under content include scope of the PROMs instrument, language used, extent of localization and contextualization. Sub-themes under administration include relevance to patients and healthcare professionals, logistics and user-friendliness of the PROMs instrument form. Table 2 shows examples of patients’ or healthcare professionals’ comments from made that map to the aforementioned themes.

**Table 2:** Narrative results of thematic analysis from Semi-structured Interviews

Main theme	Sub-theme	Narrative results	
		Patients	Healthcare professionals
Content	Scope	1. Current PROMs instruments are selectively focused and whilst providing in-depth information on i.e. symptomatology of the disease frequently neglects other important aspects like economics of treatment and psychological impact of disease.	
		1. A balance needs to be struck as to how detailed the questions should be. A significant number of PROMs questions appear repetitive and responders cannot differentiate the subtlety within.	1. PROMs instrument should also capture demographic i.e. occupation and visual requirements and comorbidity data as these factors will skew responses. 2. Not sufficient to address patient’s concerns, fears and doubts.
	Language	1. Simple and specific terms should be used in the instruments to prevent confusion, increase accuracy andreduce responder fatigue.	
		1. Is there an easier word to use besides errands	1. If technical terms are unavoidable, examples or pictures can be used to improve understanding. 2. In formulating and phrasing of PROMs questions, the usage of active voice and inclusion of a temporal comparative element will enhancement comprehension and detect disease progression.
	Localization and contextualization	1. PROMs instrument should be localized to the setting it is used and allow responders to relate the questions to their daily living.	
		1. The most relevant and useful PROMs questions are those relating to disease impact on vision and how it compromises personal safety or impair daily living. 2. Playing games such as bingo or bridge…better to use local context like playing mahjong. 3. Subway should be replaced with MRT.	
Administration	Relevance	1. The instrument needs to yield tangible benefits to the patients’ management i.e. in the form of follow up actions like referrals to be considered useful. 2. Not really relevant as I am doing good so no concerns. 3. As glaucoma is a symptomless disease in the early stages, PROMs instrument may not be particularly useful for patients with mild disease.	1. For a mild glaucoma patient, this may not be relevant
	Logistics	1. Time spend on the PROMs instrument should be keep to a minimum. Current questionnaires tend to be rather lengthy and unsuitable to be completed in a busy outpatient setting. 2. I need someone to read this out to me because I have very poor vision! 3. Traditional paper and pen administration may be impossible for those with advanced disease and poor vision.	
	User-friendliness	1. Font size, type and questionnaire layout is important to improve the user experience and enhance participation rates. This is especially so as responders are likely visual impaired due to disease or pharmacological dilation.	
		1. Cannot really see because of the dilation eyedrop…the fonts should be larger.	

### Feasibility survey results

Upon the conclusion of the semi-structured interview, all participants were presented with a simple 4 question feasibility survey. The survey questions and the results are found in Tables 3 and 4, respectively. Overall, the majority of glaucoma patients (79%) and medical professionals (94%) felt glaucoma-specific PROMs instruments had a role in the glaucoma management. Of the 5 patients who disagreed, 4 had mild glaucoma. 37% of patients and 50% of healthcare professionals felt that current PROMs instruments were not sufficiently comprehensive for clinical use. Interestingly, those who felt more had to be done for the scope of existing PROMs instruments were mainly patients at the opposite ends of the disease spectrum (4 diagnosed with mild and severe glaucoma each). Current PROMs instrument fared less well in the usability area with 54% of patients and 44% of healthcare professionals suggesting a need to improve in areas of wording and phrasing (47%), questionnaire formatting (41%) and rating methods (12%).

**Table 3:** Glaucoma specific PROMs Feasibility Survey

1.	Do you feel that such questionnaires are relevant to patients?
2.	Do you feel that such questionnaires are relevant to the healthcare team?
3.	Do you feel that current questionnaires are sufficiently comprehensive for clinical use?
4.	Do you feel that current questionnaires are sufficiently user friendly?

**Table 4:** Results of the feasibility Survey

Glaucoma specific PROMs Feasibility Survey.	Patients’ response	Healthcare professionals’ response
1. Do you feel that such questionnaires are relevant to patients?		
Yes	19 (79%)	15 (94%)
No	5 (21%)	1 (6%)
2. Do you feel that such questionnaires are relevant to the healthcare team?		
Yes	22 (92%)	15 (94%)
No	2 (8%)	1 (6%)
3. Do you feel that current questionnaires are sufficiently comprehensive for clinical use?		
Yes	15 (63%)	8 (50%)
No	9 (37%)	8 (50%)
4. Do you feel that current questionnaires are sufficiently user friendly?		
Yes	11 (46%)	9 (56%)
No	13 (54%)	7 (44%)

## Discussion

“Exploratory pilot work to assess comprehensibility, acceptability, relevance and answerability to the target population” is one step of a systematic 5-step approach to valid PROMs [25]. In our study, this step is taken to see if selected 4 PROMs have the pre-requisites for use in routine glaucoma care, negating the need for more extensive development work to create a novel PROM. To our knowledge, this is the first exploratory study that assesses the healthcare professionals’ and glaucoma patients’ perception on the content and administration of glaucoma-specific PROMs in the Singapore clinical setting.

Our results demonstrate both positive and negative aspects of glaucoma-specific PROMs for their use in the daily clinical practice. Positive aspects include the majority of healthcare professionals and patients feel that selected PROMs are relevant to patients and healthcare team. Although patients with mild glaucoma did not see the relevance of PROMs in their care, citing: “for a mild glaucoma patient, this may not be relevant” (Table 2), it can be argued that the role of PROMs in the early stage of glaucoma may still have a role in terms of assessing the impact of treatment, such as side-effects and cost incurred from treatment.

The negative aspects in our study include barriers for glaucoma-specific PROMs in routine use. Several barriers (such as logistical, social, legal, technical, and cultural barriers) have been revealed to prevent the successful embed of PROMs into the routine clinical practice [14]. Several studies have shown the attempt to collect PRO data on a large scale in the routine eye clinic setting is disappointing [27, 28]. In our study, there are concerns expressed by the participants on the user-friendliness, comprehensiveness and logistics (Tables 2 and 4). For example, the need for brevity, and the instrument should not be too long. This has also been borne out by another study looking at the Impact of Vision Impairment questionnaire, which has the best domain coverage but is poor at assessing glaucoma patients, and may be too long for routine clinical use [27]. Our participants also feel it is impossible for patients with visual impairment to self-fill PROM questionnaire in the paper format (Table 2). Browne J et al.’s study also reflects the same concern to use VF14 to assess cataract surgery [27]. The howRu instrument is effective when conducted by telephone, which may solve the potential obstacles for the PROM questionnaire used by patients with visual impairment in routine practice [29]. It would be interesting to assess glaucoma patients’ views on howRu in Singapore clinical setting in future studies.

An assessment of 11 glaucoma-specific Health-related Quality of life (HRQoL) instruments concludes that little PRO instrument covers comprehensive domains which participants are interested in [30]. Our participants also expressed a desire for a more comprehensive PROM instrument that covered more holistic issues such as financial burden of care and psychological impact of disease (Table 2). For instance, “peace of mind” concerns between 50% [31] and 80% [32] of newly diagnosed glaucoma patients, but this item has been mentioned by little glaucoma-specific PROM instruments [27]. Participants in our study reflect the similar finding that selected PROMs did “not sufficiently address patient’s concerns, fears and doubts” (Table 2). The need for inclusion of the financial impact of disease is a finding that may be unique to Singapore where patients bear a significant direct out-of-pocket burden for their care [33]. This again illustrates the need for contextual relevance when using PROMs and caution in directly “importing” PROMs that have been developed for other healthcare settings.

Our study also reveals challenges in execution that may be unique to our local context. From the outset, recruitment of willing participants proved difficult. More specifically, our recruitment is biased toward the educated participants with 54% having tertiary education (Table 1). This may be a reflection of language constraints as English was used throughout the interviews and the PROMs were all worded in English. It is noteworthy that PROMs, as a meaningful clinical routine use, must also include good generalizability to ensure that sections of the patient population are not excluded – especially the less well-educated and well-off who also tend to be more severely impacted by disease. Additionally, in our study, it is noteworthy that we recruited no Malays (Table 1), which may again reflect poor participation in this racial group due to greater comfort in using Malay as a medium of communication. Our study shows that the use of PROMs in Singapore should be available in the four main languages here, namely: English, Chinese, Tamil and Malay.

The limitations of current study should be considered. The selected PROMs may be inadequate and more work needs to be done before a large scale PROMs program can be rolled out locally. This is especially so when applied to routine care as opposed to more research-oriented settings. For a PROM to be used in the daily practice, it is important to ensure that data collection is practical and equitable, and the data produced are valid as guidelines to improve patient care and prioritize healthcare resources. Looking at the socio-demographic data from our study, it is noted that the majority participants are male (79%), while female participants is 21% (Table 1). In terms of educational status, half of participants (54%) have received the tertiary level education. There is also no Malay participant in our study. The study numbers are small to begin with and the study design qualitative in nature. However, these findings may have implications for the wider usage of PROMs in routine care. It is well-known that socioeconomic status has close correlation with the health status of an individual. Socioeconomic factors may also similarly skew response rates and response patterns such that the poorer, less educated and sicker end up being under-represented or missed altogether. In Singapore, a significant proportion of the elderly do not know English –there may be a need to have a Chinese, Indian and Malay version of the same instrument to improve generalizability. More extensive studies need to be undertaken to explore this aspect and factored into the design, not only of the instruments themselves but the logistical aspects surrounding the application of these instruments to ensure representativeness.

Despite the above limitations, we have the strength in qualitative research method-think aloud method, which is commonly used during questionnaire development to determine whether the meaning of a questionnaire item, as interpreted by the questionnaire respondent, is consistent with the questionnaire developer’s intention of that item [25]. We employed this method to garner views from participants. This method has also been used to assess glaucoma patients’ perceptions on the acceptability, relevance, comprehensibility and answerability of one glaucoma-specific PRO instrument - the Aberdeen glaucoma questionnaire [25].

## Conclusion

As the paradigm of what constitutes successful treatment shifts, PROMs will gain in prominence, and Singapore is no exception. It is heartening to know the use of glaucoma-specific PROMs is broadly welcomed by healthcare professionals and patients here. Also, our study highlights the need for caution in directly employing PROM instruments developed in other healthcare settings and the need to “contextualise” and frame these instruments for the local socio-demographic to ensure representativeness of the data collected. The identified barriers should be necessarily considered when designing and deployment of glaucoma-specific PROM instrument.
